# The Effectiveness of Lifestyle Adaptation for the Prevention of Prediabetes in Adults: A Systematic Review

**DOI:** 10.1155/2017/8493145

**Published:** 2017-04-16

**Authors:** George Kerrison, Richard B. Gillis, Shahwar I. Jiwani, Qushmua Alzahrani, Samil Kok, Stephen E. Harding, Ian Shaw, Gary G. Adams

**Affiliations:** ^1^School of Health Sciences, Faculty of Medicine South Block, Queen's Medical Centre, University of Nottingham, Nottingham NG7 2HA, UK; ^2^University of Nottingham, Sutton Bonington Campus, NCMH Building, Sutton Bonington, Leicestershire LE12 5RD, UK; ^3^Department of Food Engineering, Faculty of Engineering & Architecture, Abant İzzet Baysal University, Gölköy, 14280 Bolu, Turkey; ^4^Faculty of Medicine and Health Sciences, University Park, University of Nottingham, Nottingham NG7 2RD, UK

## Abstract

Diabetes prevalence is increasing exceptionally worldwide and with this come associated healthcare costs. The primary outcome of this systematic review was to assess glycaemic control and incidence of Type 2 diabetes mellitus (T2DM) diagnosis after exercise and dietary intervention (measured with any validated scale). The secondary outcome assessed body mass index change, weight change, and physical exercise capacity after diet and exercise intervention (measured with any validated scale). 1,780 studies were identified from searching electronic databases. Relevant studies went through a selection process. The inclusion criteria for all studies were people with prediabetes diagnosed by either impaired glucose tolerance (IGT) or impaired fasting glucose (IFG). Lifestyle adaptation reduced the incidence of diabetes development more than standard treatment. Furthermore, better glycaemic control, improved physical exercise capacity, and increased weight reduction were observed with lifestyle intervention over standard treatment. Finally, improvements over the long term deteriorated, highlighting problems with long-term adherence to lifestyle changes. Overall, cumulative incidence of diabetes is drastically reduced in the intervention groups compared to control groups (standard care). Furthermore, glycaemic control was improved in the short term, with many participants reverting to normoglycaemia.

## 1. Introduction

Cumulatively, all subcategories of diabetes mellitus affect approximately 382 million people worldwide, increasing to an estimated 592 million by 2035 [1], emphasising the global burden of diabetes. In UK, 3.2 million people have been diagnosed with diabetes; a conservative estimate predicts an increase to 5 million by 2025 [2], which equates to one in seventeen people in UK or prevalence of 6.0% [3]. In 2010/2011, the total cost to UK for diabetes was £23.7 billion, with £8.8 billion directly in T2DM costs, and total cost is predicted to rise to £39.8 billion by 2035 [4]. 10% of the NHS budget is estimated to be spent on diabetes [4, 5].

Prediabetes is a relatively recent medical term, which raises many issues for debate [6]. The American Diabetes Association [7] endorses the term, but both WHO [8] and NICE [6] oppose it. The National Institute for Clinical Excellence (NICE) considered that impaired glucose regulation (IGR) should be used instead of prediabetes; inevitable progression to diabetes is implied using prediabetes [6]. Diabetes UK (2009) states that impaired fasting glycaemia (IFG), IGT, and glycosylated haemoglobin (HbA1c) should be designated for usage between healthcare professionals, with prediabetes used for the lay person. The ADA combined IGT and IFG into the blanket term prediabetes. In 2003, the ADA lowered the threshold for IFG and HbA1c against advice from other healthcare bodies [9]. Threshold lowering was justified by needing earlier detection to reduce diabetes development and cardiovascular disease [10]. Arguments against the threshold change included consequences with life insurance, possible discrimination, and causing anxiety about developing diabetes when it might not occur [11]. Cut-off points for prediabetes are arbitrary with no biological basis for the test values [12].

The Da Qing IGT and Diabetes Study was one of the original studies on prediabetes [13] and involved 577 participants with IGT who underwent exercise and diet treatment or diet only or exercise only. Follow-up was at 2 years and 6 years to identify any participants who had developed T2DM. At 6 years, 67.7% of the control group (standard treatment) developed T2DM compared with 46.0% in the diet plus exercise group. It was assessed that the diet plus exercise intervention decreased the risk of developing diabetes by 42%. While this study was conducted nearly two decades ago, it highlights that prediabetes has always been present and can potentially be treated with diet plus exercise more effectively than the standard treatment delivered.

Oldroyd et al. [14] evaluated lifestyle modification for improving health in individuals with IGT. 39 intervention participants were encouraged to eat more fruit and vegetables, reduce fat and sugar intake, and increase dietary fibre. Furthermore, they were encouraged to achieve 20–30 minutes of aerobic activity at least once a week. At 24 months, (after commencement of study), improvements in 2 hr plasma glucose were not significantly different between the control and intervention groups. However, more participants in the intervention group compared to the control group reverted to normoglycaemia with 20% versus 13% at 24 months.

A systematic review by Orozco et al. [15] included 8 randomised controlled trials, with a total of 2241 participants using exercise plus diet treatment, compared to 2509 participants undergoing standard treatment. Interventions included calorie restriction diets that were low in fat and high in carbohydrates and fibre. Exercise interventions varied but mainly consisted of 150 minutes a week of brisk walking or other activities. The interventions were delivered by physiotherapists and dieticians across the board. On average, the incidence of diabetes reduced by 37% with exercise and diet. There were also favourable effects for body weight, waist circumference, and blood pressure. However, results of this study should be interpreted with caution as the results are now 7 years old and healthcare advances rapidly.

In 2009, Lindahl et al. tested 168 individuals for diabetes incidence, diet improvement, and exercise capacity [16]. 83 underwent 1-month intensive lifestyle intervention staying at a wellness centre where they underwent overall 140 hours of scheduled activities and had healthy meals prepared for them; 85 underwent standard treatment at home. At the 1-year follow-up, a 70% decrease in progression to T2DM was seen in the intervention group. However, at 5 years, most of the beneficial effects of the intervention had depleted; exercise capacity was one of the only outcomes which stayed at an appropriate level. This study highlights the fact that there is a maintenance problem with continuing lifestyle adaptations.

The primary outcome of this systematic review was to assess glycaemic control and incidence of T2DM diagnosis after exercise and dietary intervention. The secondary outcome assessed body mass index change, weight change, and physical exercise capacity after diet and exercise intervention (both measured with any validated scale).

## 2. Methods

**Figure 1:**
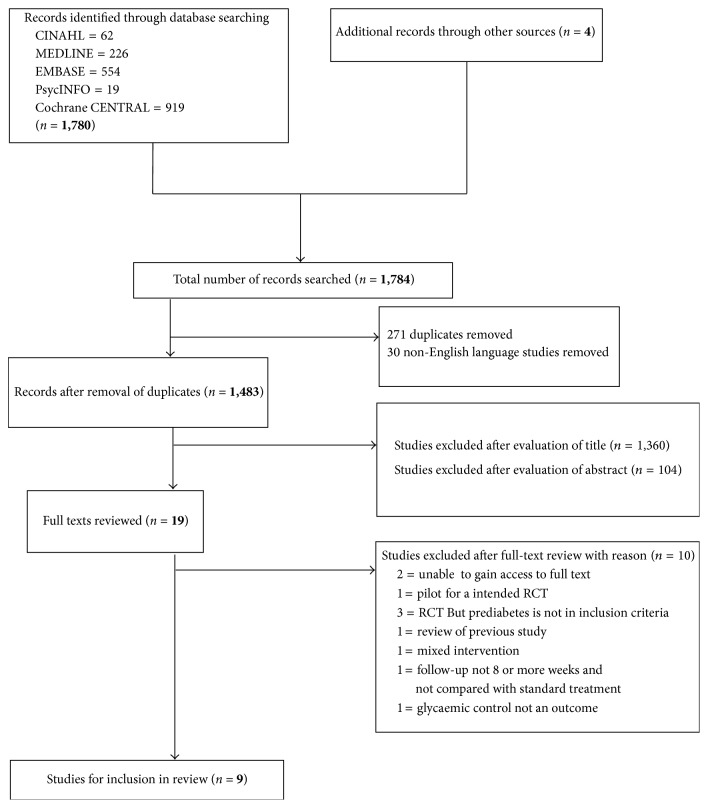
Flow chart of identification of included studies.

In total, 1,780 studies were identified from searching the electronic databases CINAHL (*n* = 62), MEDLINE (*n* = 226), EMBASE (*n* = 554), PsycINFO (*n* = 19), and Cochrane CENTRAL (*n* = 919) (Figure 1). Additional searching of reference lists from recent relevant systematic reviews and hand searching of studies identified 4 further studies. Results from the search were imported to Endnote X7 for assessment of relevance to this review. 271 duplicates and 30 studies not in the English language were removed. The remaining studies were assessed by title alone and 1,360 studies were removed. For the remaining studies, the abstract was assessed, with 104 studies deemed irrelevant. The full texts of the remaining 19 studies were examined against the inclusion and exclusion criteria for the review. 10 studies were excluded after full-text assessment. The remaining 9 studies met the inclusion criteria for the review (Table 1). All assessments were completed twice by different authors.

## 3. Excluded Studies

Of the 19 studies that had full-text review, 10 studies were excluded for not meeting the inclusion criteria. These studies were excluded for the following reasons: being unable to gain access to full text (*n* = 2), pilot for an intended RCT (*n* = 1), RCT but prediabetes is not in the inclusion criteria (*n* = 3), review of previous study (*n* = 1), mixed intervention (*n* = 1), glycaemic control not an outcome (*n* = 1), and not for specified period of 8-week intervention and not compared with normal treatment (*n* = 1).

## 4. Risk of Bias in Included Studies

**Figure 2:**
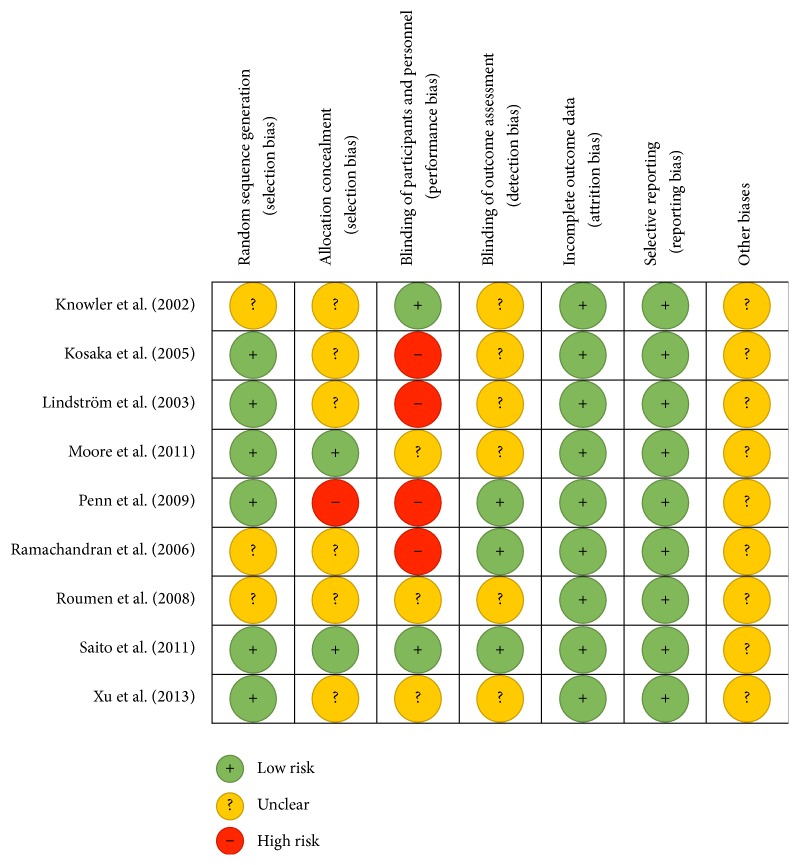
Risk of bias summary for included studies (produced in RevMan 5.3, 5 January 2015).

The methodological property of all the included studies was assessed for risk of bias (Figure 2). The assessment involved using the Cochrane collaborations tool for assessing risk of bias. The tool consists of 7 areas where bias could possibly be introduced and making judgements to assess if bias is introduced or not. This is completed by answering prespecified questions, for each of the 7 domains, and answering with either “yes” to indicate low risk or “no” to indicate high risk. “Unclear” indicates that too few details are available to make a judgement on risk of bias. The final domain, other biases, should be used to assess the study as a whole for its risk of bias. Collected data was inputted to RevMan 5.3.

All of the studies included in this review were randomised control trials (RCTs). Critical appraisal of the studies included in this review showed that the methodological quality was high to moderate for all the studies. None of the studies included a high level of bias in their design. As such, all studies were included in the review.

Random assignment to either a control or intervention group was accomplished to a low risk in 6 of the studies by randomisation lists prepared by a third party, computer generated randomisation, or random allocation tables [18–21, 24, 25].

Allocation concealment was low risk in 2 studies [20, 24], where telephone calls or numerical values were given to denote the study group. One study was high risk [21], where participants had knowledge of their allocation to the intervention group. Six studies were lacking information to make a decision on allocation concealment [17–19, 22, 23, 25]. Due to the nature of the intervention, diet, and exercise, causing changes to the participants' normal lifestyle, it is difficult to conceal allocations. Therefore, this category will not carry a large weighting in terms of selection bias for this review.

Two studies stated that they blinded participants and personnel [17, 24]. For 3 of the studies, it was unclear if the participants and personnel were blinded [20, 23, 25]. The remaining 4 studies did not blind participants or personnel [19, 22], with 2 studies specifically stating that participants are told what the intended intervention is for [18, 21].

Blinding of outcome assessment was apparent in 3 studies [21, 22, 24]. It was unclear in the remaining 6 studies whether the outcome assessors were blinded or not [17–20, 23, 25].

All of the studies had a low risk value for both incomplete outcome data and selective reporting. For other biases, it is unclear due to the limited information available on the studies. However, it is apparent that all studies maintained a similar control group and an intervention group and no results were missing from the final reports.

The country of origin for the studies varies widely. Two studies are from Japan [24] and Kosaka et al., 2002, and each of the following countries was represented by one study 1: USA [17], Finland [19], Australia [20], England [21], India [22], Netherlands [23], and China [25].

The majority of studies for exercise intervention recommended achieving and maintaining either 150 minutes of exercise a week or 30 minutes of exercise a day [17, 18, 21–23, 25]. For the remaining 3 studies, one utilised a pedometer and recommended 70,000 steps a week [24], one encouraged participants to take part in endurance and resistance training [19], and one stated “lifestyle modification” [20]. Diet interventions were targeted at motivating, encouraging, and enabling participants to achieve and maintain a target BMI or weight reduction. In one study, low glycaemic index meal replacement was used [25] and two studies did not state a target weight/BMI but provided healthy living advice [20, 22].

The delivery method for the interventions varied from questionnaires to meetings with trained medical staff, motivational phone calls, and group based sessions. All of these were for a defined period of time. For the study's outcomes, the majority of studies used the diagnosis/prognosis of diabetes as the primary or secondary outcome [17–19, 21, 22, 24, 25]. Two studies did not specifically use diagnosis of diabetes as an outcome; these studies, as well as some others, focused on plasma glucose concentration [20, 23]. Other outcomes from the studies included changes in BMI, waist circumference, physical activity capacity, calorie intake, and diabetes knowledge.

## 5. Characteristics of Participants

**Figure 3:**
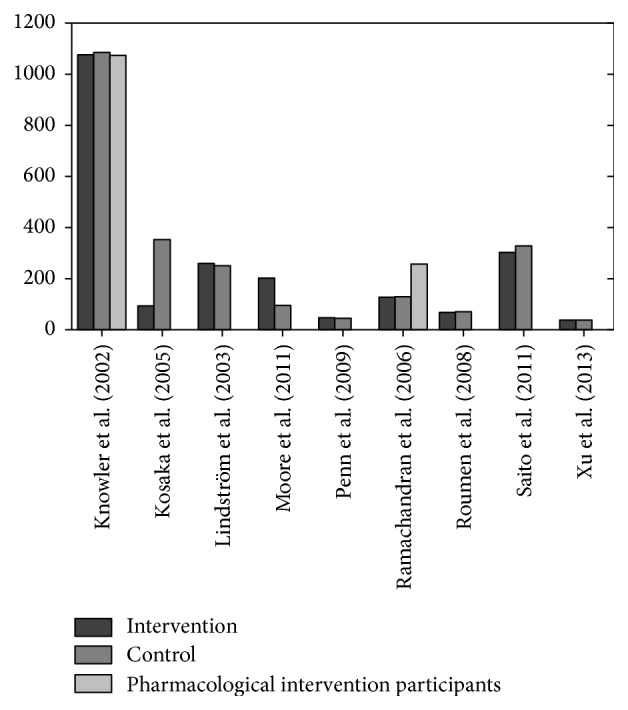
Sample size of included studies.

**Figure 4:**
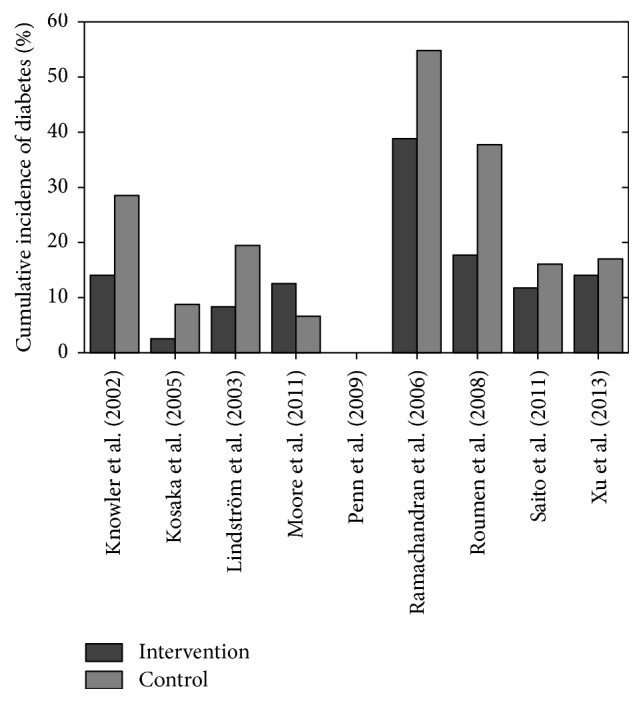
Cumulative incidence of diabetes for control and intervention groups.

The characteristics of participants are summarised in Table 2. The study sample size (adjusted by removal of pharmacological interventions) ranged from 88 to 2,161 in size, with overall 4,695 participants involved in either lifestyle intervention or control groups (Figure 3). The total participants were 6,022 with pharmacological arms of studies included: 249 in Ramachandran et al. [22] and 1,078 in Knowler et al. [17].  Figure 4 expresses the breakdown for intervention, control, and pharmacological intervention participants for relevant studies.

All of the studies included both male and female participants. Apart from Kosaka et al.'s study [18] that had male participants only, it was acknowledged in the text that a previous study completed by the researcher included both males and females but there was a high dropout rate for female participants so male-only study was completed this time. The study by Ramachandran et al. [22] stipulated that only Asian Indian participants would be included in the study; all other studies were open to all ethnicities; however, coincidentally some only had one ethnicity included. The mean age of participants ranged between 46.1 ± 5.7 and 62.5 ± 10.1. The mean body mass index (BMI) of participants ranged from 23.8 ± 2.1 to 34.1 ± 5.5. It should be noted that 4 of the studies are from Asian countries that have a lower BMI cut-off for BMI being recognised as a risk factor for diabetes; hence, some studies have a low BMI at baseline.

The inclusion criteria for all studies were prediabetes diagnosed by either IGT [17, 19–23] or IFG [18, 24, 25]. All studies, apart from Lindström et al.'s study [19], stated a numerical value for diagnosis of prediabetes. The study by Lindström et al. states that prediabetes diagnosis is in accordance with the WHO [8] criteria for diagnosis, previously outlined in this review. Some of the studies had extra inclusion criteria which included age restrictions of participants [17, 19, 21, 24, 25] and BMI above a certain threshold [17, 19, 21, 25].

Exclusion from all studies was due to diagnosis of diabetes before or during the study. Diagnosis of diabetes was completed by testing either fasting plasma glucose concentration or glucose concentration 2 hours after a 75 g oral glucose load. All studies, apart from Lindström et al.'s [19] and Xu et al.'s [25] studies, state a numerical value for cut-off for diabetes diagnosis. This means that the reviewing author is unable to specify what method was used by Lindström et al. [19] and Xu et al. [25] to diagnose diabetes. Further exclusions for some studies were due to chronic illness that seriously reduced life expectancy or ability to partake in physical activity and medications that affect glucose concentration, mental illness, malignant neoplasm, and a range of kidney, liver, heart, and pancreas diseases.

## 6. Characteristics of Interventions

The characteristics of the study interventions are summarised in Table 3. All of the included studies promoted healthy eating and an increase in moderate physical activity. The delivery method varied in procedure and duration. The majority of studies used face-to-face individual interview style information, delivered by a dietician or a physiotherapist [17–19, 21, 23]. The remaining studies used either group based sessions carried out by a trained facilitator [20], utilised phone calls to provide information that coincided with 6 monthly face-to-face follow-up meetings [22], used a pedometer to self-motivate participants to increase exercise with face-to-face meetings [24], or after initial educational lectures on diet and exercise were given 3 months of meal replacements [25]. The duration of intervention ranged from 6 months to 5 years; the majority of studies followed up participants for 36 months.

Three of the studies included offering further interventions. Lindström et al. [19] offered voluntary group sessions, low-fat cooking lessons, visits to local supermarkets, and between-visits phone calls and letters. Penn et al. [21] offered discount cards of 80% to physical exercise facilities and personal trainer sessions. Finally, Roumen et al. [23] had participants participate 3 times a year in an exercise programme using a heartbeat watch. Two of the studies included pharmacological interventions in separate arms [17, 22]; in these cases, and for the purposes of this review, the results of these arms are not reported.

All of the studies had weekly, monthly, or quarterly consultations with the participants to review blood glucose concentrations, weight, BMI, and food diaries among other individual study outcomes.

## 7. Primary Outcome: Incidence of Diabetes

The primary outcomes for this review are the incidence of diabetes development and glycaemic control.  Table 4 summarises the primary outcome date. For each study, the baseline characteristics were assessed to have no significant differences between control and intervention groups which would affect the overall outcomes. Roumen et al. [23] had an age difference of 2 years between the control and intervention groups, the control group's mean age was 54 years and the intervention group's mean age was 52 years. The reviewing author judged this to be an insignificant difference.

**Figure 5:**
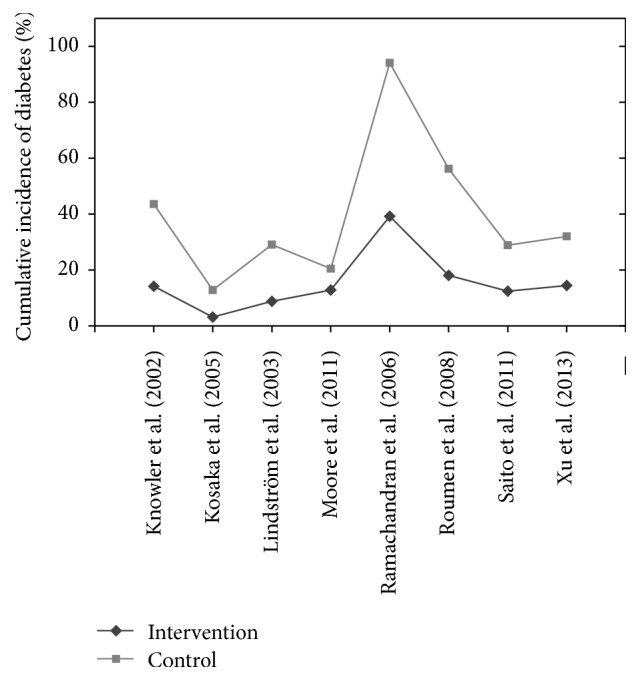
Cumulative incidence of diabetes across studies examined.

Eight studies provided information for the cumulative incidence of diabetes for control and intervention groups. Penn et al. [21] provided information on the cumulative incidence difference between the intervention and control groups and not on each individual group.  Figure 5 shows the cumulative incidence of diabetes breakdown for each study.

Cumulative incidence of diabetes is higher in the control group than in the intervention group in all cases, apart from Moore et al.'s study [20]. The cumulative incidence for diabetes in the intervention and control groups, respectively, is as follows: Knowler et al. [17], 14.4% and 28.9%; Kosaka et al. [18], 3.0% and 9.3%; Lindström et al. [19], 9.0% and 20.0%; Moore et al. [20], 13.0% and 7.0%; Ramachandran et al. [22], 39.3% and 55%; Roumen et al. [23], 18.0% and 38.0%; Saito et al. [24], 12.2% and 16.6%; and Xu et al. [25], 14.6% and 17.5%. Penn et al. [21] had a cumulative incidence difference of diabetes diagnosis of 55% less in the intervention group compared to the control group.

Figure 5 shows the range of results for the intervention and control groups. The cumulative incidence of diabetes ranged from 3.0% [18] to 39.3% [22] for the intervention group, with a mean value of 15.44% (excluding [21]) across 8 studies. The cumulative incidence of diabetes for the control group ranged from 7.0% [20] to 38.0% [22], with a mean value of 24.01%.

Two studies report incidence of diabetes in people-years. Knowler et al. [17] report that, in the intervention group, there were 4.8 cases per 100 people-years and, in the control group, there were 11.0 cases per 100 people-years. Penn et al. [21] had 32.7 cases per 1000 people-years for the intervention group and 67.1 cases per 1000 people-years for the control group.

## 8. Primary Outcome: Glycaemic Control

Four studies [17, 21, 22, 24] do not use glycaemic control as either a primary or a secondary outcome. The remaining studies reported glycaemic control in a number of ways, ranging from improvement in oral glucose tolerance test (OGTT) [18] to glycaemic values staying within prediabetes range [20], change from prediabetes to normoglycaemia [20, 25], and 2-hour plasma glucose levels to show positive or negative change from baseline [19, 20, 23, 25].

Kosaka et al. [18] reported OGTT improvement from baseline to end of study at 4 years. Improvement of 53.8% and 33.9% was observed for the intervention group and the control group, respectively. Moore et al. [20] reported cumulative incidence of prediabetes of 45% for intervention group and 67% for control group at the end of the study. Two studies reported change from prediabetes to normoglycaemia. Moore et al. [20] had results of 43% for the intervention group and 26% for control group. Xu et al. [25] reported results of 39% for intervention group and 7.5% for control group.

The four studies that reported 2-hour plasma glucose levels represented data by showing actual glycaemic levels of difference in glycaemic levels from baseline to the end of the study. Two studies showing actual glycaemic levels had results. Roumen et al. [23] had intervention group's results as follows: baseline, 8.59 ± 0.24, 1 year, 7.96 ± 0.29, and 3 years, 8.55 ± 0.32; and they had control group's results as follows: baseline, 8.46 ± 0.23, 1 year, 8.83 ± 0.29, and 3 years, 9.35 ± 0.33. Moore et al. [20] had intervention group's results as follows: baseline, 8.47 ± 1.39, and 6 months, 7.79 ± 2.31; and they had control group's results as follows: baseline, 8.08 ± 1.78, and at 6 months, 7.98 ± 2.68. Two studies used difference in glycaemic control levels. Xu et al. [25] had intervention group's results as follows: baseline, 8.90 ± 1.25, with change at 1 year of −1.24 ± 0.35; and they had control group's results as follows: baseline, 9.24 ± 1.58, with change at 1 year of +0.85 ± 0.86. Lindström et al. [19] had intervention group's results as follows: baseline, 8.9 ± 1.5, change at 1 year, −0.9 ± 1.9, and change at 3 years, −0.5 ± 2.4; and they had control group's results as follows: baseline, 8.9 ± 1.5, change at 1 year, −0.3 ± 2.2, and change at 3 years, −0.1 ± 2.2. For the studies that reported glycaemic control, the intervention groups had vastly more improvements in 2-hour plasma glucose concentration, glycaemic levels reverting to normal, and less participants remaining with prediabetes compared to the control groups.

## 9. Secondary Outcome: Physical Exercise Capacity

The results of the secondary outcomes are summarised in Table 5. Four of the 9 studies reported on physical exercise capacity by meeting recommended goals, maximum oxygen volume (VO_2_), or leisure time and other time physical activities. The 5 other studies either did not report physical activity levels or did not report sufficient details about them to be included in the results.

Knowler et al. [17] reported how many intervention group participants were meeting 150 minutes of physical activity a week. The study had intense input up to the 24th week mark. At this point, 74% of participants were completing 150 minutes of physical activity a week. The level of adherence dropped to 58% by the end of the study. Levels for the control group were not reported.

Lindström et al. [19] reported that moderate to vigorous leisure time activity increases more significantly in the intervention group compared to the control group. Results were reported in minutes per week of exercise. The control group at baseline was achieving 21 minutes, increasing marginally to 23 minutes at the end of the study. Intervention group's baseline results were 16 minutes, increasing to 50 minutes per week by the end of the study.

Ramachandran et al. [22] did not report specific figures for physical exercise adherence; a graph is provided but it is difficult to ascertain specific values from this. The study does state that adherence levels changed from 41.7% at baseline to 58.8% at the end of the study for the intervention group.

Roumen et al. [23] reported both VO_2_ (ltr/min) max and days where 30 minutes of activity was met. The VO_2_ level for the intervention group at baseline was 2.22 ± 0.61, changing at 1 year by +0.13 ± 0.25, at 2 years by +0.10 ± 0.25, and at 3 years by +0.05 ± 0.35. The control group's max VO_2_ at baseline was 2.13 ± 0.55, changing at 1 year by +0.02 ± 0.21, at 2 years by −0.05 ± 0.23, and at 3 years by −0.06 ± 0.21. The number of days the target of 30-minute activity was being met changes for the intervention group by +0.89 ± 2.75 days and for the control group by −0.55 ± 3.31 days.

## 10. Secondary Outcome: Change in BMI

 Four studies reported change in BMI. Moore et al. [20] reported BMI change for the intervention group from 29.66 ± 5.33 at baseline to 28.72 ± 5.00 at 6 months. The change for the control group was from 29.76 ± 4.94 at baseline to 29.50 ± 5.24 at 6 months. These values indicate decreases of 0.94 for the intervention group and 0.29 for the control group.

The 3 remaining studies reported difference in BMI from baseline at different stages of the studies. Lindström et al. [19] had baseline results of 31.4 ± 4.5 for the intervention group, changing at 1 year by −1.6 ± 1.8 and at 3 years by −1.3 ± 1.9. They had baseline results of 31.1 ± 4.5 for the control group, changing at 1 year by −0.4 ± 1.3 and at 3 years by −0.3 ± 2.0. Roumen et al. [23] reported baseline results of 29.6 ± 3.8 for the intervention group, changing at 1 year by −0.94 ± 1.25, at 2 years by −0.61 ± 1.49, and at 3 years by −0.36 ± 1.47. They had baseline results of 29.2 ± 3.3 for the control group, changing at 1 year by −0.20 ± 1.39, at 2 years by −0.02 ± 1.17, and at 3 years by +0.08 ± 1.80. Finally, Xu et al. [25] had baseline results of 26.80 ± 3.13 for the intervention group, changing at 1 year by −0.66 ± 0.13. They had baseline results of 25.72 ± 3.83 for the control group, changing at 1 year by −0.22 ± 0.15.

## 11. Secondary Outcome: Change in Weight

All of the studies reported on changes in mean weight (kg) lost over the study periods. Ramachandran et al. [22] states no significant weight change for intervention group. The 8 remaining studies either provided the baseline and end-of-study mean weights or provided the difference observed from baseline to the end of the study.

Moore et al. [20] states that the baseline data for the intervention group was 80.7 ± 16.01 and at the end of the study it was 78.11 ± 14.98. The control group had baseline data of 82.02 ± 16.27 and at the end of the study it was 81.20 ± 17.39.

Four studies reported mean weight change from baseline to the end of the study. Knowler et al. (2008) had a mean weight difference of −5.6 kg for the intervention group and −0.1 kg for the control group. Kosaka et al. [18] had mean weight change of −2.18 kg for the intervention group and −0.39 kg for the control group. Penn et al. [21] had change of −2.3 kg for intervention group and change of +0.01 kg for the control group. Finally, Saito et al. [24] had weight change of −2.5 kg and −1.1 kg for the intervention group and the control group, respectively.

The remaining 3 studies reported their findings in more detail. Lindström et al. [19] had baseline results of 86.7 ± 14.0 for intervention group, changing after 1 year by −4.5 ± 5.0 and at 3 years by −3.5 ± 5.1. They had baseline results of 85.5 ± 14.4 for control group, changing after 1 year by −1.0 ± 3.7 and at 3 years by −0.9 ± 5.4. Roumen et al. [23] reported baseline results of 87.5 ± 13.7 for intervention group, changing at 1 year by −2.77 ± 3.69, at 2 years by −1.76 ± 4.34, and at 3 years by −1.08 ± 4.30. They had baseline results of 83.0 ± 11.7 for control group, changing at 1 year by −0.62 ± 3.92, at 2 years by −0.22 ± 3.26, and at 3 years by +0.16 ± 4.91. Finally, Xu et al. [25] had baseline results of 68.24 ± 9.73 for intervention group, changing at 1 year by −1.75 ± 0.35. They had baseline results of 69.69 ± 1.36 for control group, changing at 1 year by −0.55 ± 0.40.

Three of the studies further reported how many participants met the weight loss goal target. Knowler et al. (2008) had a weight loss target of ≥7%; at 24 weeks (end of intense input) 50% of the intervention group had achieved this. This figure dropped at the end of the study to 38% of participants. Lindström et al. [19] had target weight loss of ≥5%. At 1 year, 46% of the intervention group and 14% of the control group had achieved this target. Saito et al. [24] had target weight loss of ≥5%; 32% of the intervention group and 18% of the control group achieved this level.

## 12. Discussion

### 12.1. Incidence of Diabetes

The results advocate lifestyle intervention to be utilised for effectively delaying or preventing the progression of prediabetes to T2DM. All of the studies, besides Moore et al.'s study [20], had a reduced incidence of diabetes with lifestyle intervention being adopted in comparison to the control (normal treatment). These findings correlate with previous studies assessing whether lifestyle interventions could conceivably be adopted to reduce the incidence of diabetes [13, 15, 26–28].

De Vegt et al. [29] found that, over a span of 6.4 years, 34% of their study subjects, with baseline IGT, naturally developed T2DM. Similarly, Meigs et al. [30] found that 21% of their study subjects, with baseline IFG or IGT, progressed naturally to T2DM during a 5-year study. Seven of the 9 studies had diabetes incidence rates for the intervention groups below Meigs et al.'s [30] and De Vegt et al.'s [29] findings. Determining lifestyle intervention reduced the likelihood of T2DM diagnosis more than natural progression. Two studies were above the natural progression threshold [21, 22]. Penn et al. [21] did not report cumulative incidence of diabetes, so comparison was unavailable. Ramachandran et al. [22] had an intervention group's cumulative incidence rate for diabetes of 39.3%; this was a spike when compared to other studies (Figures 4 and 5). However, study participants were native Asian Indians who are physiologically more susceptible to diabetes due to their ethnicity [31, 32]. The natural progression studies were not completed on such high risk groups. Furthermore, Ramachandran et al.'s [22] control group had diabetes incidence rate of 55%, showing that lifestyle intervention reduced the incidence of diabetes compared to the control.

Moore et al.'s study [20] was the only one whose control group had a lower incidence of diabetes than the intervention. This was also the only study to use group based sessions to instruct the intervention group. This tentatively indicates that group based approach may not be as effective as one-to-one sessions for lifestyle advice. Interventions need to be individualised and performed by a skilled healthcare professional trained in the specific field. The results obtained in this study may be due Moore et al. focusing on reducing diabetes. However, incidence of diabetes reduction for the intervention group was similar to the other included studies. Furthermore, follow-up was only for 6 months; longer follow-up might affect the results.

### 12.2. Glycaemic Control

Five studies reported glycaemic control as a primary or secondary outcome. Results revealed that a considerable proportion of participants in the intervention groups reverted to normoglycaemia at an increased rate compared to the control group. Furthermore, significant reductions in 2-hour plasma glucose concentrations were present with lifestyle intervention in the short term. Likewise, there was a reduction in 2-hour plasma glucose levels for the control groups; however, levels are substantially less significant.

Results indicated obstacles associated with long-term maintenance of improved glycaemic regulation. Two studies reported glycaemic control at 3 points [19, 23]: baseline, 1 year, and 3 years. For both studies, mean glycaemic concentration increased at year 3 compared to year 1 after the initial drop from baseline observed at year 1. These findings were from two studies only, so they should be interpreted with caution. Overall, results suggested that lifestyle intervention in the short term had an undeniable positive effect for glycaemic control but long-term maintenance problems. Different individuals require different motivational input to achieve behavioural changes; this could range from identification of prediabetes at baseline to continuous support to change behaviours. Findings by Norris et al. [33] and Yoon et al. [34] correlate with this review's findings, supporting the effectiveness of lifestyle adaptation in the short term but with long-term adherence complications.

All included studies had intensive initial period for study intervention. After this, face-to-face contact reduced in frequency. Improvements in short-term glycaemic control may be associated with the initial intense intervention. When intensity dropped, so did glycaemic control. Kim and Oh [35] found that an intervention group with intense healthcare input, via regular phone call contact, had better lifestyle adherence and consequently improved glycaemic control than a control group with standard healthcare input. These findings correlate with those of the review. However, in contrast, Radhakrishnan [36] found that standard care may be as effective as tailored individualised care for self-management behaviours in long-term conditions. The motivational effect of intense input needs to be assessed separately and considered in future RCTs.

Improvements in glycaemic control could have been attributed to some of the study subjects volunteering and already having willingness to change [37]. This is likely to lead to subsequent changes in lifestyle, which will benefit the individual's glycaemic control. For lifestyle interventions to work, individuals need the initial willingness or intention to change so they are prepared to persevere with lifestyle modifications over the long term [38].

### 12.3. Physical Exercise Capacity

Physical exercise capacity was reported in 4 studies. Improvements in exercise capacity for the intervention groups were more significant than for the control group. Although increases in physical exercise capacity were observed, the levels remain low for participants achieving 150 minutes of activity a week. Herbst et al. [39] found in adolescents with T2DM that over half of the participants did not perform regular physical activity. This is not just the case for T2DM; it is apparent with other long-term conditions. Serour and Alqhenaei [40] found that individuals at risk of cardiovascular disease did not complete exercise programmes, even though they were fully aware of the positive effects exercise has on their condition.

Physical exercise capacity at the end of the intense intervention period compared with the end of the trial deteriorated; however, it improved from baseline. There was high exercise capacity in the short term, which reduced as intervention intensity did. This was also found to be the case in Woodard and Berry [41] and Madden et al.'s [42] works. Furthermore, Tran et al. [43] found that, after the diabetes aerobic and resistance exercise (DARE) trial, only 41% of participants completed regular exercise 8 to 12 years after an intensive 6-month trial. A positive correlation exists between glycaemic control and physical exercise capacity. Exercise only trials are needed to assess how exercise affects glycaemic control.

Exercise and motivational problems were observed. Tulloch et al. (2013) found that continuous intervention with exercise specialists is needed to maintain exercise levels. Furthermore, Visram et al. [44] found that newly diagnosed diabetics had fears and lack of understanding about exercise which acted as a barrier. In terms of the practical implications of this study, the assessment of self-efficacy in people with prediabetes may need to be the first step in the development of individualised lifestyle interventions and additional booster sessions to improve long-term exercise capacity [46].

### 12.4. Weight and BMI

In 8 studies, lifestyle intervention significantly reduced BMI and weight, implying that lifestyle intervention involving diet and exercise is an effective treatment for weight reduction for adults with prediabetes. These results are not surprising as lifestyle adaptation has previously been recognised as effective for weight and BMI reduction with improvements in glycaemic control also identified [47, 48].

The same pattern that was present with exercise capacity and glycaemic control is evident with weight and BMI change. Over the short term, there was significant improvement in weight. However, over the long term, both weight and BMI increased. The results are in line with those of Norris et al. [49] and Kouvelioti et al. [50] in two separate systematic reviews of weight loss.

Weight and BMI regain occurred in the intervention group but levels still remained significantly below the baseline results, whereas, for some cases in the control groups, weight regain above the original baseline results was observed. Even with modest weight loss, every kilogram of weight loss is associated with a 16% reduction in diabetes risk [51] and has long-lasting effects for T2DM risk reduction [52]. The results from the review suggest that small percentages of sustained weight change are beneficial in reducing the risk of prediabetes progression to T2DM and improving glycaemic control.

## 13. Conclusion

This systematic review aimed to assess the feasibility of lifestyle interventions being used to treat prediabetes and enhance glycaemic control. The outcome measures were to assess cumulative incidence of diabetes development, glycaemic control, physical exercise capacity, and changes in weight/BMI. Nine RCTs met the inclusion criteria from a total of 1,784 relevant studies searched. The review provides evidence for the effectiveness of lifestyle interventions to treat prediabetes. Overall, cumulative incidence of diabetes is drastically reduced in the intervention groups compared to control groups (standard care). Furthermore, glycaemic control was improved in the short term, with many participants reverting to normoglycaemia. In the long term, glycaemic control diminished, but glycaemic control was still superiorly better managed than baseline results and control groups. A similar scenario is true for weight and BMI, where short-term reductions are replaced by long-term weight and BMI increases. Physical exercise capacity improved at an increased rate in the intervention groups compared to the control groups; however, it is still significantly lower than the recommended 150 minutes of exercise a week.

As a result of the findings from this review, lifestyle intervention should be provided as a treatment option for adult prediabetes patients to improve glycaemic control and reduce the prospect of their condition developing into type 2 diabetes mellitus. However, before this is implemented in a practice setting, more research needs to be completed to assess how motivation to change can be maintained over the long term. Furthermore, RCTs with large participant numbers, completed in the UK, need to be undertaken to assess the generalizability of lifestyle intervention treatments for patients accessing care from the NHS.

## Figures and Tables

**Table 1:** Summary for characteristics of included studies.

#	Study	Country	Study setting	Sample size	Characteristics of exercise intervention	Characteristics of diet intervention	Outcome
(1)	Knowler et al. [17]	USA	27 medical centres	3234	Engage in moderate activity exercise for 150 minutes a week.	Achieve and maintain weight reduction of 7% initial body weight. Low-calorie and low-fat diet.	P = diagnosis of diabetes. S = physical activity, weight change.

(2)	Kosaka et al. [18]	Japan	Toranomon Hospital	458 (male only)	Achieve or maintain moderate exercise (e.g., 30 min bike ride or 30–40 min walking daily).	Achieve and maintain a BMI of 22. Advice on food alternatives and diet given.	P = development of diabetes. S = glycaemic control and changes in weight.

(3)	Lindström et al. [19]	Finland	Helsinki, Kuopio, Turku, Tampere, and Oulu health centres	522	Endurance and resistance training.	Behavioural changes. Recommends 0.5–1 kg weight loss per week.	P = physical activity and weight loss. S = glycaemic control and diabetes development.

(4)	Moore et al. [20]	Australia	2 urban areas and 1 rural area of Victoria, Australia	307	States lifestyle modification.	States lifestyle modification	P = diabetes knowledge, dietary and exercise adherence. S = glycaemic control, BMI, waist circumference.

(5)	Penn et al. [21]	England	Royal Victoria Infirmary, Newcastle upon Tyne	102	Physical activity equivalent to 30-minute moderate activity every day.	Reduce saturated fat intake to <30%. Increase energy from carbohydrates to >50%, increase fibre intake, and achieve weight loss to reach target BMI of 25	P = diagnosis of diabetes. S = participation in physical activity and diet changes.

(6)	Ramachandran et al. [22]	India	Various service organisations	531	Walk briskly 30 minutes a day.	Advice on healthy eating.	P = diagnosis of diabetes. S = body weight and waist circumference.

(7)	Roumen et al. [23]	Netherlands	Maastricht, Netherlands	147	30 minutes a day five days a week. 3 times a year participation in activity wearing a heartbeat watch.	5–7% body weight loss, based on Dutch guidelines for healthy eating.	P = glycaemic control. S = body weight, maximal aerobic capacity.

(8)	Saito et al. [24]	Japan	38 hospitals and clinics across Japan	641	Pedometers with self- monitoring of goals (recommended goal of 70,000 steps a week).	5% reduction in body weight. Restricting excess intake of fat and carbohydrates. Self-goals to increase healthy diet.	P = diagnosis of diabetes.

(9)	Xu et al. [25]	China	2 health centres in urban areas of Shanghai	88	Recommended moderate exercise, for example, 30–40-minute walking a day	Daily breakfast replacement with low glycaemic index food for first 3 months of study. Advice on healthy eating.	P = glycaemic control and diagnosis of diabetes. S = body weight, BMI.

P: primary outcome; S: secondary outcome; BMI: body mass index (kg/m^2^).

**Table 2:** Summary of characteristics of participants.

#	Study	Sample size (T, I, C)	Mean age (years)	Mean BMI (kg/m^2^) at baseline	Inclusion criteria	Exclusion criteria	Blood glucose concentration for diagnosis of prediabetes	Cut-off for diagnosis of diabetes
(1)	Knowler et al. [17]	T = 3234 I = 1079 C = 1082 Total of I and C = 2161	Overall = 34.0 ± 6.7 I = 50.6 ± 11.3 C = 50.3 ± 10.4	Overall = 34.0 ± 6.7 I = 33.9 ± 6.8 C = 34.2 ± 6.7	≥25 years old, BMI ≥ 24, IGT	Diabetes, taking glucose altering medication, illness that seriously reduces life expectancy, or ability to participate in the trial	2 hours after 75 g oral glucose load 7.8 mmol/L to 11.0 mmol/L	≥11.1 mmol/L 2 hours after 75 g oral glucose load. ≥7.0 mmol/L fasting plasma glucose

(2)	Kosaka et al. [18]	Men only T = 458 I = 102 C = 356	Mean age not stated	I = 24.0 ± 2.3 C = 23.8 ± 2.1	IFG	Diabetes, malignant neoplasm, suspected or diagnosed disease of liver, pancreas, endocrine organs, or kidneys, ischaemic heart disease, or cerebrovascular disease	2 hours after 75 g oral glucose load 7.8 mmol/L to 11.0 mmol/L	Fasting plasma glucose of ≥7.8 mmol/L

(3)	Lindström et al. [19]	T = 522 I = 265 C = 257	I = 55 ± 7 C = 55 ± 7	I = 31.4 ± 4.5 C = 31.1 ± 4.5	40–64 years old, BMI ≥ 25, IGT	Diabetes	States based on WHO criteria	Level for diagnosis not stated

(4)	Moore et al. [20]	T = 307 I = 208 C = 99	Overall = 62.5 ± 10.1	I = 29.66 ± 5.33 C = 29.79 ± 4.94	IGT	Diabetes	2 hours after 75 g oral glucose load 7.8 mmol/L to 11.0 mmol/L	>11.0 mmol/L 2 hours after 75 g oral glucose load. ≥7.0 mmol/L fasting plasma glucose

(5)	Penn et al. [21]	T = 102 I = 51 C = 51	Overall = 57.2 ± 1.7	I = 34.1 ± 5.5 C = 33.5 ± 4.6	≥40 years old, BMI ≥ 25, IGT	Diabetes, chronic illness that meant being unable to participate in moderate physical activity, special diet for medical reasons	2 hours after 75 g oral glucose load 7.8 mmol/L to <11.1 mmol/L	≥11.1 mmol/L 2 hours after 75 g oral glucose load

(6)	Ramachandran et al. [22]	T = 531 I = 133 C = 136 Total of I and C = 269	I = 46.1 ± 5.7 C = 45.2 ± 5.7	I = 25.7 ± 3.3 C = 26.3 ± 3.7	IGT	Diabetes	2 hours after 75 g oral glucose load 7.8 mmol/L to 11.0 mmol/L	Fasting plasma glucose of ≥ 7.8 mmol/L

(7)	Roumen et al. [23]	T = 147 I = 74 C = 73	I = 54.2 ± 5.8 C = 58.4 ± 6.8	I = 29.6 ± 3.8 C = 29.2 ± 3.3	IGT	Diabetes, chronic illness, medication known to change glucose concentrations	2 hours after 75 g oral glucose load 7.8 mmol/L to <12.5 mmol/L	States based on WHO (1999) criteria

(8)	Saito et al. [24]	T = 641 I = 311 C = 330	Median age only stated at: I = 50 (44–54) C = 48 (41–54)	I = 26.9 ± 2.6 C = 27.1 ± 2.6	30–60 years old, BMI ≥ 24, IFG	Diabetes, ischaemic heart disease, stroke, chronic nephritis, pituitary disease, thyroid disease, adrenal gland disease, mental illness, gastrectomy, malignant tumour, medication effecting glucose concentrations	Fasting plasma glucose 5.6 mmol/L 6.7 mmol/L	≥11.1 mmol/L 2 hours after 75 g oral glucose load. ≥7.0 mmol/L fasting plasma glucose

(9)	Xu et al. [25]	T = 88 I = 46 C = 42	I = 60.35 ± 9.78 C = 56.55 ± 8.61	I = 26.80 ± 3.13 C = 25.72 ± 3.83	≥25 years old, BMI ≥ 18.5, IFG	Diabetes, stroke, CHD, malignancies in past 5 years, suspected disease of liver, pancreas, or kidney	2 hours after 75 g oral glucose load 7.8 mmol/L to 11.0 mmol/L	Diabetes cut-off not stated

T: total; I: intervention; C: control; IGT: impaired glucose tolerance; IFG: impaired fasting glucose; BMI: body mass index (kg/m^2^); WHO: World Health Organization.

**Table 3:** Summary of interventions.

#	Study	Organisation of exercise and dietary intervention and who it is delivered by	Duration of intervention	Organisation of control and who it is delivered by	Duration of control	Duration of participant follow-up
(1)	Knowler et al. [17]	16 lessons for the first 24 weeks of enrolment. One-to-one basis covering diet, exercise, and behavioural modifications. Subsequent individual and group sessions monthly. Delivered by: case manager	16 sessions over first 24 weeks and then monthly for average of 2.8 years	Written information given to participants at a 1-year annual 30-minute individual session which emphasised the importance of healthy living. Participants encouraged using the food pyramid and a set out diet. Delivered by: case manager	Average of 2.8 years	Average of 2.8 years (range: 1.8 to 4.6 years)

(2)	Kosaka et al. [18]	Participants to weigh themselves at least once a week and reduce weight. The following advice was repeated every 3-4 months at hospital visits: dietary; fat intake; reduce alcohol intake; reduce snacks and increase physical activity. Delivered by: hospital staff	4 years	Advised on meal portion size reduction and to increase physical activity levels. Objectives repeatedly explained every 6 months. Delivered by: hospital staff	4 years	4 years

(3)	Lindström et al. [19]	7 face-to-face consultations in the first year, lasting 30 minutes to 1 hour, at weeks 0, 1-2, and 5-6 and then at months 3, 4, 6, and 9. Subsequent meetings were once every 3 months. Aimed to individualise diet recommendations for each participant and goal setting. Dietician encouraged increase in physical endurance and resistance activities at each meeting. Participants offered voluntary group sessions, low-fat cooking lessons, visits to local supermarket and between-visits phone calls and letters. Delivered by: nutritionist	7 sessions in first 9 months and then one session every 3 months for 3 years	Given general information about lifestyle and diabetes risks. Delivered in one-to-one or groups session, lasting 30 minutes to 1 hour Control group given same information on weight reduction and physical activity as intervention, but consultations were not individualised. Delivered by: nutritionist	1 session at initiation of study, participants carried on control for 3 years	3 years

(4)	Moore et al. [20]	The healthy living course. Sessions with groups of 6 to 10 participants. Aim to promote healthy lifestyle. 6 sessions over 6 months providing information on diet, exercise, motivation, goal setting, and stress. Delivered by: facilitators who undertook 3-day training workshop	6 months	Waiting list	6 months	6 months

(5)	Penn et al. [21]	Individual sessions for 30 minutes per session, immediately following randomisation and 2 weeks later and then monthly for first 3 months and every 3 months thereafter for up to 5 years. Discount card of 80% to physical exercise facilities and personal trainer sessions. Delivered by: dietician and physiotherapist	Up to 5 years	Offered health promotion advice including widely available written leaflets on healthy eating and physical activity	Up to 5 years	Average of 3.1 years (range: 0–5 years)

(6)	Ramachandran et al. [22]	Advice on healthy eating and regular physical exercise given by monthly phone calls for first 6 months. Individual sessions delivered once every 6 months. Delivered by: dietician, doctor, and social worker	3 years	States “given standard healthcare advice.” Delivered by: dietician, doctor, and social worker	1 session at initiation of study, participants carried on control for 3 years	3 years

(7)	Roumen et al. [23]	Every 3 months, a 1-hour counselling session on individualised dietary advice and increasing physical activity. 3 times a year participants participated in an exercise programme using a heartbeat watch. Delivered by: dietician and physiotherapist	3 years	Briefly informed about the beneficial effects of a healthy diet and physical activity, with no individual advice provided. Delivered by: dietician	1 session at initiation of study, participants carried on control for 3 years	3 years

(8)	Saito et al. [24]	Given pedometers and general information on diabetes and lifestyle modification. 9 follow-up sessions at 1, 3, 6, 12, 18, 24, 30, and 36 months to set goals for next meeting and encourage weight loss and moderate exercise. Delivered by: medical staff (nurses, dieticians, physiotherapists, and doctors)	36 months	4 sessions at 12-month intervals starting at 0 months. Provided instructions on physical activity and weight loss voluntarily without follow-up support. Delivered by: medical staff (nurses, dieticians, physiotherapists, and doctors)	36 months	36 months

(9)	Xu et al. [25]	Educational lecture on balanced diet, regular exercise, and behavioural strategies to control blood glucose. To follow 2007 Chinese guidelines for the management of type 2 diabetes and dietary guidelines for Chinese. Daily meal replacement for 3 months of intervention. Given individualised diet instructions and recommended moderate exercise. Delivered by: not specified	3 months	Educational lecture on balanced diet, regular exercise, and behavioural strategies to control blood glucose. To follow 2007 Chinese guidelines for the management of type 2 diabetes and dietary guidelines for Chinese. Delivered by: not specified	1 initial session	12 months

**Table 4:** Summary of primary outcomes.

#	Study	Baseline data	Diabetes development	Glycaemic control
(1)	Knowler et al. [17]	Baseline characteristics for intervention and control had no significant difference.	Diabetes incidence: 4.8 cases per 100 person-years in the intervention group and 11.0 cases per 100 person-years in the control group. Cumulative incidence of diabetes was 14.4% for the intervention group and 28.9% for the control group at the end of the study period.	No primary or secondary outcome

(2)	Kosaka et al. [18]	Baseline characteristics for intervention and control had no significant difference.	Cumulative incidence of diabetes was 3.0% for intervention group and 9.3% for control group. The development of diabetes reduced by 67.4% in the intervention group compared to the control group.	At the end of 4-year study, improvement in OGTT was 53.8% for intervention and 33.9% for control

(3)	Lindström et al. [19]	Baseline characteristics for intervention and control had no significant difference.	Cumulative incidence of diabetes was 9% for intervention group and 20% for control group.	2 h plasma glucose *Intervention*: baseline = 8.9 ± 1.5, 1 year = −0.9 ± 1.9, and 3 years = −0.5 ± 2.4. *Control*: baseline = 8.9 ± 1.5, 1 year = −0.3 ± 2.2, and 3 years = −0.1 ± 2.2

(4)	Moore et al. [20]	Baseline characteristics for intervention and control had no significant difference.	Cumulative incidence of diabetes was 13% for intervention group and 7% for control group.	Cumulative incidence of prediabetes at the end of study was 45% for intervention and 67% for control. Moving from prediabetes to nondiabetes was 43% for intervention and 26% for control. 2 h plasma glucose *Intervention*: baseline = 8.47 ± 1.39 and at 6 months = 7.79 ± 2.31. *Control*: baseline = 8.08 ± 1.78 and at 6 months = 7.98 ± 2.68

(5)	Penn et al. [21]	Baseline characteristics for intervention and control had no significant difference.	Diabetes incidence of 32.7 per 1000 person-years of follow-up in intervention group and 67.1 per 1000 person-years of follow-up in control group. The overall cumulative incidence of diabetes was 55% less in the intervention group compared to the control group.	Glycaemic control not a primary or secondary outcome

(6)	Ramachandran et al. [22]	Baseline characteristics for intervention and control had no significant difference.	Cumulative incidence of diabetes was 39.3% for intervention group and 55% for control group. Number of people who would need to be treated to prevent one case of diabetes in the intervention group was 6.4.	Glycaemic control not a primary or secondary outcome

(7)	Roumen et al. [23]	Age was higher in the control group than intervention: 54 years and 52 years, respectively. No other significant differences.	Cumulative incidence of diabetes was 18% for intervention group and 38% for control group.	2 h plasma glucose *Intervention*: baseline = 8.59 ± 0.24, at 1 year = 7.96 ± 0.29, and at 3 years = 8.55 ± 0.34. *Control*: baseline = 8.46 ± 0.23, at 1 year = 8.83 ± 0.29, and at 3 years = 9.35 ± 0.33

(8)	Saito et al. [24]	Baseline characteristics for intervention and control had no significant difference.	Cumulative incidence of diabetes was 12.2% for intervention group and 16.6% for control group.	Glycaemic control not a primary or secondary outcome. States that the fasting plasma glucose or 2 h plasma glucose levels significantly decreased more in intervention group than in control group

(9)	Xu et al. [25]	Baseline characteristics for intervention and control had no significant difference.	Cumulative incidence of diabetes was 14.6% for intervention group and 17.5% for control group.	Reverting to normal glucose levels at the end of study was 39.0% for intervention and 7.5% for control

OGTT: oral glucose tolerance test.

**Table 5:** Summary of secondary outcomes.

#	Study	BMI change (kg/m^2^)	Weight change (kg)	Physical exercise capacity
(1)	Knowler et al. [17]	Not primary or secondary outcome of the study	50% of the intervention group achieved ≥7% weight loss by 24 weeks. At the end of the study this was 38% Average weight loss was 5.6 kg in intervention and 0.1 kg in control	Activity levels of 150 minutes a week met by 74% of intervention group at 24 weeks. This reduced to 58% by the end of the study

(2)	Kosaka et al. [18]	Not primary or secondary outcome of the study	In intervention group body weight decreased by 2.5 kg after 1 year and then increased thereafter but remained 2.18 kg lower than baseline at 4 years. Control group's weight decreased by 0.39 kg by end of 4 years	Not primary or secondary outcome

(3)	Lindström et al. [19]	BMI at baseline for intervention = 31.4 ± 4.5, at 1 year = −1.6 ± 1.8, and at 3 years = −1.3 ± 1.9. BMI at baseline for control = 31.1 ± 4.5, at 1 year = −0.4 ± 1.3, and at 3 years = −0.3 ± 2.0	Target to lose ≥ 5% weight achieved by year one: 46% in intervention and 14% in control. Weight for intervention at baseline = 86.7 ± 14.0, at 1 year = −4.5 ± 5.0, and at 3 years = −3.5 ± 5.1. Weight for control at baseline = 85.5 ± 14.4, at 1 year = −1.0 ± 3.7, and at 3 years = −0.9 ± 5.4	Moderate to vigorous leisure time activity increased in the intervention group compared to the control group. Intervention group at baseline (min/week): mean of 16 minutes and increased to mean of 50 minutes. Control group at baseline (min/week): mean of 21 minutes and increased to mean of 23 minutes.

(4)	Moore et al. [20]	BMI at baseline for intervention = 29.66 ± 5.33 and at 6 months = 28.72 ± 5.00. BMI at baseline for control = 29.79 ± 4.94 and at 6 months = 29.50 ± 5.24	Weight for intervention at baseline = 80.7 kg ± 16.01 and at 6 months = 78.11 ± 14.98. Weight for control group at baseline = 82.02 ± 16.27 and at 6 months = 81.20 ± 17.39	Not primary or secondary outcome

(5)	Penn et al. [21]	Not primary or secondary outcome of the study	Change in mean weight at 1 year for intervention and control groups was −2.3 kg and +0.01 kg, respectively	There were no significant differences in sustained physical activity levels

(6)	Ramachandran et al. [22]	Not primary or secondary outcome of the study	No significant difference in weight seen with intervention group and significant increase in weight for control group (figure not provided estimated at 0.7 kg increase from graph provided)	Physical activity adherence showed an improvement of 41.7% to 58.8% in intervention group from baseline to the end of study

(7)	Roumen et al. [23]	Mean BMI for intervention from baseline = 29.6 ± 3.8, 1 year = −0.94 ± 1.25, 2 years = −0.61 ± 1.49, and 3 years = −0.36 ± 1.47. Mean BMI for control from baseline = 29.2 ± 3.3, 1 year = −0.20 ± 1.39, 2 years = −0.02 ± 1.17, and 3 years = +0.08 ± 1.80	Mean weight (kg) change for intervention from baseline of 87.5 ± 13.7, 1 year = −2.77 ± 3.69, 2 years = −1.76 ± 4.34, and 3 years = −1.08 ± 4.30. Mean weight change for control from baseline of 83.0 ± 11.7, 1 year = −0.62 ± 3.92, 2 years = −0.11 ± 3.26, and 3 years = +0.16 ± 4.91	In the intervention group, the number of days where at least 30 minutes of physical activity was achieved increased by 0.89 ± 2.75 days from baseline, while in the control group the number of days decreased by −0.55 ± 3.31. VO_2_ (l/min) max at baseline for intervention = 2.22 ± 0.61; this changed at 1 year = +0.13 ± 0.25, 2 years = 0.10 ± 0.25, and 3 years = 0.05 ± 0.35. VO_2_ max for control at baseline = 2.13 ± 0.55; this changed at 1 year = +0.02 ± 0.21, 2 years = −0.05 ± 0.23, and 3 years = −0.06 ± 0.21

(8)	Saito et al. [24]	Not primary or secondary outcome of the study	Achieving weight loss of 5% or more at 36 months = 32% for intervention and 18% for control. Mean weight reduction at 36 months = 2.5 kg for intervention and 1.1 kg for control	Not primary or secondary outcome

(9)	Xu et al. [25]	BMI for intervention at baseline = 26.80 ± 3.13 and at 1 year = −0.66 ± 0.13. BMI for control at baseline = 25.72 ± 3.83 and at 1 year = −0.22 ± 0.15	Weight loss for intervention at baseline was 68.24 ± 9.73 and changed at 1 year to −1.75 ± 0.35. Weight loss for control at baseline was 69.69 ± 10.36 and changed at 1 year to −0.55 ± 0.40	Not primary or secondary outcome

BMI: body mass index (kg/m^2^); VO_2_: oxygen volume (ltr/min).
